# Overview of NIA Budget

**DOI:** 10.1093/geroni/igab046.1044

**Published:** 2021-12-17

**Authors:** Richard Hodes

**Affiliations:** National Institute on Aging, Bethesda, Maryland, United States

## Abstract

Dr. Hodes will discuss budget and overall research priorities for the National Institute on Aging.

